# Loving-Kindness Meditation to Target Affect in Mood Disorders: A Proof-of-Concept Study

**DOI:** 10.1155/2015/269126

**Published:** 2015-06-01

**Authors:** Stefan G. Hofmann, Nicola Petrocchi, James Steinberg, Muyu Lin, Kohki Arimitsu, Shelley Kind, Adriana Mendes, Ulrich Stangier

**Affiliations:** ^1^Department of Psychological and Brain Sciences, Boston University, 648 Beacon Street, Boston, MA 02215, USA; ^2^Department of Psychology, University of Frankfurt, Germany

## Abstract

Conventional treatments for mood disorders primarily focus on reducing negative affect, but little on enhancing positive affect. Loving-kindness meditation (LKM) is a traditional meditation practice directly oriented toward enhancing unconditional and positive emotional states of kindness towards oneself and others. We report here two independent and uncontrolled studies carried out at different centers, one in Boston, USA (*n* = 10), and one in Frankfurt, Germany (*n* = 8), to examine the potential therapeutic utility of a brief LKM group intervention for symptoms of dysthymia and depression. Results at both centers suggest that LKM was associated with large-sized effects on self-reported symptoms of depression (*d* = 3.33 and 1.90), negative affect (*d* = 1.98 and 0.92), and positive affect (*d* = 1.63 and 0.94). Large effects were also found for clinician-reported changes in depression, rumination and specific positive emotions, and moderate effects for changes in adaptive emotion regulation strategies. The qualitative data analyses provide additional support for the potential clinical utility of the intervention. This proof-of-concept evaluation of LKM as a clinical strategy warrants further investigation.

## 1. Introduction

A number of studies carried out throughout the 1990s and early 2000s have shown that mindfulness based treatments can be effective for a number of psychological problems, especially for treating mood and anxiety disorders [1–4]. These mindfulness practices typically encourage present-moment awareness, curiosity, and openness to sensorial experiences while focusing attention on breathing [3, 5]. Popular and promising contemporary mindfulness-based interventions include mindfulness-based stress reduction (MBSR; e.g., [6, 7]) and mindfulness-based cognitive therapy (MBCT; e.g., Kuyken et al. [8]) for treating mood disorders, as well as various physical and psychological problems. Although the mechanism of these treatments is still not well understood, a recent review of the literature suggests that these interventions work through moderating cognitive and emotional reactivity and reducing rumination and worrying [9].

Other traditional but less studied Buddhist practices include loving-kindness meditation (LKM) and compassion meditation, which are exercises oriented toward enhancing unconditional, positive emotional states of kindness and compassion [10]. Loving-kindness (*metta* in Pali), which is derived from Buddhism, refers to a mental state of unselfish and unconditional kindness to all beings [11]. When practicing LKM, the person gently repeats certain phrases in order to direct a positive energy of feeling, called* metta*, towards other people, as well as oneself. Metta refers to a mental state of unselfish and unconditional kindness to all beings. The phrases are not used as a mantra that loses its meaning with repetition. Rather, the phrases are intended to keep one's attentional focus on metta and the target of it. Therefore, the phrases are used mindfully each time, bringing one's full awareness to the phrases, their meaning, and the feelings they bring up.

In the Buddhist tradition, LKM is typically combined with other meditation practices, especially sensorial and breathing-focused mindfulness meditation. The aim of LKM is to develop an affective state of unconditional kindness to all living creatures (for a review, see [12–14]). This meditation practice is believed to broaden attention, enhance positive emotions, and lessen negative emotional states. It is believed to shift a person's basic view of the self in relation to others and increase empathy [15].

In traditional Buddhist practices, LKM is considered particularly helpful for people who have a strong tendency toward hostility or anger toward others or themselves (e.g., [16, 17]). Therefore, LKM may be particularly useful for improving positive affect and reducing negative affect, such as anxiety and mood symptoms in clinical populations [12]. However, very few studies have examined this issue, despite some indirect evidence supporting this notion. For example, it has been shown that even a brief (7-minute) exercise of LKM was sufficient to induce changes with small to medium effect sizes in implicit and explicit attitudes toward strangers [18]. Moreover, LKM has been shown to enhance a person's daily experiences of positive emotions, which, in turn, increases personal resources that hold positive consequences for the person's mental health [19]. The study by Fredrickson et al. [19] examined employees of a large business software and information technology services company. Participants either received LKM or were assigned to a waitlist control group. LKM was taught during six 60-minute group sessions conducted over 7 weeks with 20–30 participants and one instructor per group. The results showed that LKM led to significant shifts in people's daily experiences of a wide range of positive emotions, including love, joy, gratitude, contentment, hope, interest, pride, awe, and amusement. These increases in positive emotions could be observed both within the trajectories of change in daily emotions over the span of 9 weeks and also 2 weeks after the formal training had ended. Although the shifts in positive emotions were relatively small in magnitude, they were associated with increases in a variety of personal resources, including mindful attention, self-acceptance, positive relations with others, and good physical health over the course of 9 weeks. Moreover, the gains in personal resources led participants to become more satisfied with their lives and to experience less depressed mood. This study supports an earlier finding [20] showing that an 8-week LKM program for chronic low back pain led to significantly greater improvement in mood than standard care.

Very few studies have investigated LKM [21, 22] or related interventions (i.e., self-compassion; [23–25]) in clinical or clinical analog samples. An uncontrolled pilot study by Johnson and colleagues [22] examined the effects of 6 weekly 1-hour LKM courses as an intervention for treating negative symptoms in schizophrenia. A total of 18 outpatients were enrolled in the study, and 2 patients did not complete at least 50% of the sessions, leaving 16 posttest completers. The authors reported that participants showed significant improvements on self-report measures of positive emotions and decreases in overall negative symptoms, specifically anhedonia. However, the data have to be interpreted with caution given the very small number of patients, the uncontrolled study design, and the lack of blind assessment.

Another recent pilot study by Kearney and colleagues [21] examined a 12-week LKM training for 42 veterans with posttraumatic stress disorder in an open pilot trial. Measures of traumatic symptoms, depression, and mindfulness were obtained at baseline, after the 12-week intervention, and 3 months follow-up. Attrition was low with 74% of subjects completing the intervention. The study reported a large effect size for trauma symptoms and a medium effect size for depression at 3-month follow-up.

These early findings are promising and support the notion that LKM might be well suited for reducing negative affect and enhancing positive affect in individuals with emotion dysregulation [12]. This is consistent with results from experimental studies suggesting that LKM decreases anxiety and stress [20], positively influences emotional responses to neutral stimuli [18], and promotes positive emotions, such as trust, love, hope, and compassion [19]. Despite this literature, traditional psychotherapy for depression has been primarily focused on decreasing negative affect, whereas strategies to enhance positive affect are rarely considered [26]. Although positive and negative affects are negatively associated, the absence of negative affect does not necessarily lead to enhanced positive affect [27]. We recently presented an emotion dysregulation model of mood disorders, suggesting that depression is associated with a dysregulation of negative affect, combined with a deficiency in positive affect [27]. This model leads to the hypothesis that one effective way to treat mood disorder is to decrease negative affect and increase positive affect using LKM.

Our aim was to conduct a proof-of-concept evaluation about the feasibility and efficacy of a stand-alone LKM training (i.e., without any cognitive, behavioral, or any other established therapeutic procedures) to target symptoms of depression, as well as negative and positive affects. We will report the results of two independent, small-scaled, and uncontrolled clinical trials conducted at different centers using similar interventions in individuals with dysthymic symptoms (Study 1) and persistent depressive disorder (Study 2). We hypothesized that the interventions would lead to significant improvements in subjects' depressive symptoms, negative affect, and an increase in positive affect. We supplemented the quantitative data analyses with qualitative analyses in a subset of participants from both samples. Although both studies shared the same main objectives, they were conducted independently but concurrently using different populations and facilitators/therapists with very little communication between the two sites.

## 2. Study 1

Study 1 was conducted at the Center for Anxiety and Related Disorders at Boston University. The purpose of this study was twofold. First, our aim was to develop a 12-session group intervention solely based on LKM techniques (i.e., without any other specific treatment factors, such as cognitive restructuring and behavioral activation). The second objective was to test this treatment protocol in a sample of individuals with symptoms of dysthymia. Our hypotheses were that LKM leads to (1) an improvement in symptoms of depression; (2) a decrease in negative affect; and (3) an increase in positive affect. We further examined the effects of the intervention on changes in rumination.

## 3. Method

### 3.1. Intervention

The intervention was conducted in groups with 6–8 participants and 2 facilitators/therapists per group. The groups generally met once a week for 12 successive weeks and each session was approximately 60 minutes long. Each session began with guided meditations that involved various meditation exercises. The exercises combined a discussion of the principles of LKM and of how to incorporate mindfulness and LKM into daily life and activities.

The structure and content were similar to the structure used by Kearney and colleagues [21] who applied this technique to PTSD. As part of the intervention, participants were taught the basics of sitting mindfulness and how to concentrate their thoughts and feelings on the present moment in a nonjudgmental fashion. In session 3, participants were introduced to the LKM strategies. As part of these strategies, participants were taught to identify and focus the positive feelings they have toward a benefactor who has helped them before. In session 4, participants were instructed to focus on positive feelings when they are around someone or something they deeply care about (but not a romantic partner). In later sessions, participants were instructed to transfer these feelings to themselves, to a neutral individual, to a difficult person (e.g., somebody who had inflicted pain), and finally to all living beings. After each session, participants were instructed to practice the LKM for 15–20 min/day during the week.

More specifically, the session-by-session treatment outline with the primary topics covered during the classes were as follows: session 1: introduction to principles of mindfulness; session 2: practice of sensorial meditation (sitting and breathing meditation); session 3: introduction to LKM; practice of LKM with focus on a benefactor who the participant holds gratitude for; session 4: continuing LKM, adding focus on a beloved one (one's child, mother, etc.); session 5: continuing LKM, adding focus on self (as noted, this was sometimes moved to a later session for individuals who found focus on self too challenging); session 6: continuing LKM, adding focus on a neutral person; session 7: continuing LKM, adding focus on a difficult person; sessions 8–11: continuing LKM, gradually moving to all living beings; and session 12: LKM to all human beings and wrap-up.  Table 1 provides an overview of the session structure.

Of note, we occasionally had to change the order of the LKM steps. During a typical LKM meditation practice, the focus on the self happens at an early stage, before focusing on a neutral or difficult person. However, many participants found it more challenging to focus on positive feelings towards self than towards a neutral or even difficult person. Therefore, the order had to be rearranged such that the focus on one's self occurred after focusing on the neutral and sometimes even after the difficult person.

### 3.2. Participants

Participants were recruited from the greater Boston metropolitan area via online resources (e.g., Craigslist), board postings at a local university, and flyers posted on community bulletin boards at public places (e.g., cafes, restaurants and subway stations). A brief description of the study was also posted online with a phone number, email address, and the dysthymic disorder symptoms screening question,* Have you been feeling depressed or in a low mood for most of the day, more days than not, nearly every day for about 1 year?* When the research coordinator was contacted by a participant, the coordinator set up a date and time for the potential participant to meet with a member of the study staff at the Center for Anxiety and Related Disorders at Boston University to obtain informed consent.

Study criteria included: The person responds positively to the question: “*Have you been feeling depressed or in a low mood for most of the day, more days than not, nearly every day for 1 year or longer*?”, a negative affect scale score of the Positive and Negative Affect Schedule (PANAS; [28]) of at least 21.6 (1 SD above the mean of normative sample), and 18 years of age or older. Exclusion criteria included a Beck Depression Inventory-II (BDI-II; [29]) score of 30 or higher (more than moderate depression), having imminent suicide risk, receiving any psychiatric or psychological treatment for a mood or anxiety disorder at the time of the assessment, and meeting diagnostic criteria for bipolar disorder or schizophrenia assessed by the Anxiety Disorders Interview Schedule for DSM-IV (ADIS-IV; [30]).

A total of 113 participants were screened to participate in this open trial. Thirty-one (27%) of them met screening criteria and were invited to an initial assessment. Of these, 6 potential participants declined this visit and 4 were assessed but not enrolled in the study because they could not make time for the training.

A total of 21 participants were enrolled in the training. The sample primarily consisted of males (62%). The mean age was 37.90 (SD = 13.71, range 20–73). Slightly more than half of the participants (57%) identified themselves as white; 19% were Hispanic/Latino, 14% black or African American, 14% Asian, and 14% “other.” Most of the participants (67%) were single or never married; 19% were married or lived with partner. The majority (81%) reported that they had completed at least some college education; 33% had a full-time job, and 19% had a part-time job. Of the 21 participants, 10 (48%) completed the intervention, whereas 11 (52%) terminated before the last session, resulting in 10 treatment completers.

### 3.3. Measures

After obtaining written consent, participants were administered the BDI-II [29], the PANAS [28], the Rumination Response Scale (RRS; [31]), and the Dispositional Positive Emotions Scale (DPES; [32]). The same instruments were administered again at the end of the intervention.

## 4. Results of Study 1

### 4.1. Acceptability

Seven participants dropped out during the first three sessions, possibly due to lack of motivation. Four additional participants dropped out after the fourth session because of work-related reasons (*n* = 2), relocation (*n* = 1), and preference for individual psychotherapy (*n* = 1). Participants who did not complete the study were not distinguishable from completers on any baseline psychological variables (*p*'s > 0.1). After completing the intervention, participants were asked to evaluate the program by answering three questions. First, they were asked what the most enjoyable/helpful part of the intervention was. Most of them identified meditation in session (*n* = 5), followed by homework meditation (*n* = 2) and sharing their experiences with others (*n* = 1). Second, they were asked what was the least enjoyable/helpful in the training. Participants reported the following: nothing (*n* = 5) and some parts of meditation (*n* = 2). Third, they were asked in what way the intervention influenced their daily lives. Most participants (*n* = 8) noted that they experienced positive affect (e.g., kindness, comfort, connectedness, or openness) to a greater degree than before. There were no unexpected adverse events over the course of the study.

### 4.2. Effect of Intervention

The changes in outcome variables for the 10 completers are shown in Table 2. Repeated measures *t*-tests using the pre-post-scores showed that participants reported a large-sized and significant increase and a large-sized and significant decrease in the negative subscale of the PANAS. The BDI-II showed a very large (*d* = 3.33) decrease over the course of the intervention. Finally, the results of the DPES suggested a large-sized and significant increase in the subscales joy, contentment, love, pride, amusement, and awe. Finally, the RRS showed a large-sized and significant decrease.

## 5. Discussion of Study 1

The findings of Study 1 suggest that the 12-session LKM intervention was associated with a large-sized increase in positive affect as measured by the PANAS and more positive emotions as measured by the DPES. This was consistent with our prediction that LKM leads to an increase in positive affect. In addition, this intervention was associated with a strong decrease in negative affect and rumination, as well as a very large reduction in BDI scores (Cohen's *d* = 3.33). The major limitation of this proof-of-concept study is the small sample size, lack of a control group, and the very high attrition rate at the beginning of the intervention. Thus although the intervention appears to have strong effects on positive and negative affects, it might not be suitable as a stand-alone intervention and more appropriate as an additional component in a conventional CBT protocol. Despite the relatively high attrition rate, participants who decided to complete the intervention generally reported a very positive experience with the treatment. In an attempt to examine the consistency of these findings, we conducted another small-scale and independent study carried out in a different setting and location.

## 6. Study 2

Study 2 was conducted at the Department of Clinical Psychology and Psychotherapy at Frankfurt University. The aim of this pilot study was to test a treatment protocol that was based on the protocol of Study 1 in a sample of patients with chronic depression (persistent depressive disorder). In order to lower the attrition rate, the treatment also included components patients might expect from a treatment targeting depressive symptoms, including general group discussions about depression and breathing exercises to reduce distress. We expected that the group program would (1) decrease the symptoms of depression, (2) decrease negative affect, and (3) increase positive affect. In addition, we examined the effects of the intervention on emotion regulation strategies.

## 7. Method

### 7.1. Intervention

A similar but not identical LKM intervention was employed as in Study 1. Some of the differences had to do with the session schedule. Moreover, the rationale for the intervention provided to the participants was adapted to the specific patient sample. More specifically, patients were told that the intervention would target suppression of negative affect as well as deficits in experiencing positive affect that contribute to the maintenance of depression. Finally, this study included a relatively higher proportion of sensorial mindfulness meditation than in Study 1.

The intervention was again conducted in a group format, this time consisting of 9 sessions, each lasting approximately 2 hours, and conducted within 8 consecutive weeks. Twelve people participated in the group sessions with 6–8 participants and 2 facilitators/therapists in each group. Each session began with guided mindful meditations, followed by the discussion of weekly homework practice. Beginning with session 5, the focus of the group program was on LKM. After introducing the concept, LKM exercises were practiced in group sessions and difficulties with the exercises were discussed. Furthermore, formal exercises and informal practices of mindfulness and LKM in daily life were given as homework. Session 5 was followed by a 4-hour retreat session, within the same week.

More specifically, the outline of the intervention was as follows: session 1: introduction to principles of mindfulness meditation, raisin exercise, and introduction to body scan; session 2: practice of body scan; session 3: practice of sitting and breathing meditation; session 4: sitting meditation and 3-minute breathing space; session 5: introduction to LKM; focus on benefactor and friend; session 6 (retreat): continuing LKM, adding focus on self; session 7: continuing LKM, adding focus on neutral person; session 8: continuing LKM, adding focus on difficult person; session 9: continuing LKM, moving to all living beings. It should be noted that, in order to adjust the treatment to this specific population of chronically depressed individuals, the LKM training was more flexible than in Study 1 and focused on depressive symptoms. Whereas the order of the metta exercises was constant for participants in Study 1, as outlined in Table 1, it was more flexible in Study 2 and developed in collaboration with the patient.

### 7.2. Participants

Participants were recruited from the outpatient clinic of the department, via the department website and through advertisement in a local newspaper. Participants who were not recruited from the outpatient clinic underwent a telephone screening interview, which covered the major symptoms of depression, the duration of current depression, and possible comorbid disorders. Twenty-four individuals who appeared eligible for the study were invited to a diagnostic interview and assessed for eligibility.

Potentially eligible participants underwent a* Structured Clinical Interview for DSM-IV* Axis I and II (SCID-I and II, German Version; [33]), the* Hamilton Rating Scale for Depression* (HRDS; [34]) and the* Psychiatric Status Rating*, modified for* Persistent Depressive Disorder* according to DSM-5 (PSR-PDD; [35]; adapted by [36]). The interviews and clinical ratings were conducted by independent clinicians trained in these procedures.

Inclusion criteria were the following: (1) a primary diagnosis of* Persistent Depressive Disorder* (DSM-5) and (2) 18–70 years of age; the exclusion criteria included the following: current addictions or ongoing substance abuse, acute or past manic or psychotic symptoms, PTSD, obsessive compulsive disorder, eating disorders, odd/dramatic personality disorders, acute suicidality, and severe medical conditions, concurrent psychotherapy.

Twelve people were selected for the study; seven were female and five male. The mean age was 52.08 (SD = 10.23, range 36–70). The mean age at first episode of depression was 22.60 years (SD = 7.40). Three of the patients took antidepressants and continued to take it during the meditation program; one of them was able to withdraw from benzodiazepines during treatment.

### 7.3. Measures

After obtaining written consent, participants completed the Beck Depression Scale (BDI-II, German version), the Hamilton Rating Scale for Depression (HRDS; [34]), the Affective Style Questionnaire (ASQ; [37]), the Positive and Negative Affect Scale (PANAS), and the German version of the Response Styles Questionnaire (RSQ-D; [38]). The same instruments were administered again at the end of the intervention.

## 8. Results of Study 2

### 8.1. Acceptability

Four patients dropped out during the first four sessions. The reasons reported by the patients were dissatisfaction with the program (*n* = 2), preference for individual psychotherapy (*n* = 1), and intervertebral disk surgery (*n* = 1). At the end of the intervention, the eight participants who completed the treatment were asked to evaluate the components of the program on a five-point Likert scale, ranging from 0 (not helpful) to 4 (very helpful). Participants rated the meditation program in general to be helpful (M = 2.86, SD = 1.07), with mindfulness (M = 2.71, SD = 0.71) and LKM (M = 2.50, SD = 1.29) being evaluated as the most beneficial. Among the individual exercises, sitting mediation (M = 3.00, SD = 0.58), body scan (M = 2.86, SD = 1.07), breathing space (M = 2.71, SD = 1.11), and informal mindful exercises (M = 2.57, SD = 0.98) were evaluated as most helpful. The ratings for individual LKM exercises ranged from moderately beneficial when focusing on a* benefactor* (M = 2.43, SD = 1.27),* friend* (M = 2.29, SD = 1.11), and* neutral person* (M = 2.00, SD = 1.29) but less beneficial when focusing on the* self* (M = 1.50, SD = 1.05) and a* difficult person* (M = 1.14, SD = 1.07).

### 8.2. Effect of Intervention

The changes in the outcome variables for the 8 completers are presented in Table 3. The intervention resulted in a large-sized decrease in the BDI-II, a large-sized decrease in the HRDS and the PANAS negative affect subscale, and a large-sized increase in the PANAS positive affect subscale. The effect size for the change in rumination was between medium and large. Changes in emotion regulation were only medium-sized and limited to the adjusting and accepting subscales.

## 9. Discussion of Study 2

The findings of Study 2 were largely consistent with the results of Study 1. As in Study 1, the intervention was associated with a large-sized effect on depression. This was true for both the self-rated and the clinician-rated scales of depression. These results provide further support for the potential therapeutic value of LKM for mood disorders.

It should be noted that the total number of LKM exercises in Study 2 was smaller than those of Study 1, which might have been the reason why the changes in depression and positive affect were not as large in this study as in Study 1. However, the differences in results might also be due to the differences in the sample characteristics and higher symptom severity of participants in Study 2 as compared to Study 1. These issues need to be addressed in future and controlled studies with carefully diagnosed patients.

## 10. Qualitative Data Analyses of Study 1 and Study 2

To provide a qualitative evaluation of the LKM program, we asked the following questions: (1) Is LKM an acceptable intervention to the participants? (2) Do participants derive any benefit from LKM? (3) Are the participants satisfied with the meditation program? In order to answer these questions, we adopted a qualitative data analytic approach using a random subset of participants from Study 1 and Study 2.

## 11. Method

Data were gathered from eight random participants, four of them from Study 1 at Boston and four from Study 2 at Frankfurt. The participants were interviewed after completing the intervention. The characteristics of the participants are depicted in Table 4.

### 11.1. Data Collection and Analysis

Interviews were conducted by two staff members in Boston and Frankfurt, based on semistructured interviews with using the same preformulated and open-ended questions. More specifically, the interviews included the following questions:

1. What comes to mind when you think about the past 12 weeks of the mediation program?
2. What experiences did you gain with the exercises? Did you use any of the exercises you learned from the meditation program in your daily life?
3. How did you feel in the group?
4. How would you describe the changes in your mental state since the beginning of the program?
5. To what extend have your expectations being satisfied?

Each interview was recorded, transcribed, and analysed. Following the recommendation by Mayring [39], we conducted a qualitative content analysis.

## 12. Results

Consistent across sites, participants' answers fell into a number of broad categories. Below, we provide some illustrative examples of participants' reports, most of which were consistent across the two study sites in Boston (B) and Frankfurt (F); see Table 4.

### 12.1. Motivation and Expectation

This category describes the range of prior motivational aspects and expectations that participants expressed about their expectations and reasons to participate in these studies.

#### 12.1.1. Relief from Depression

The majority of participants noted that finding relief from depression-related symptoms was a major motivating factor. For example, one participant (F2) said: “Before the study got started, I was feeling bad (…) actually very bad. If I did not feel bad, I was at best in a neutral state.” Another participant (F1) noted: “[I am in] self-denial, [have] lack of self-esteem; I'd rather kill myself (…) and never feel my sad life [again].” Another participant (B1) reported: “I've always struggled with what they're calling chronic low mood. I was on anti-depressants for a long time. They helped, but I didn't want to be on them and I've been off them for several years now. I wanted a different solution.”

#### 12.1.2. Curiosity and Openness to Experience

When participants were asked about their initial expectations and how they felt after completing the intervention, most of them reported that the program resulted in greater openness to new experiences. For example, one participant (F4) stated: “I did not have high expectations, I was simply lucky to find something that helps me to move forward in another direction.”

#### 12.1.3. Disappointment with Past Treatments

Other participants had no particular expectations but were feeling disappointed with past treatments. For example, one participant noted: “I'd rather have no expectations, but I just faced anything new the meditation program might bring. I have done so many things, and when I had expectations before, I most often failed. [I was] pushing through everything that did not help, and [that] was wrong (…) but here, I had the feeling that I have a benefit; from the first session, I was happy to participate in the study” (F1).

### 12.2. Perceived Benefits

All participants reported that they developed an ability to relate differently to their emotions and thoughts.

#### 12.2.1. New Relationship to Negative Thoughts and Emotions

Participants described a nonjudgmental attitude and an increased sense of control over negative thoughts and emotions. For example, one participant said: “I find that I benefitted from it just from the sheer fact of being aware, of paying attention to my moods and my feelings. So I could step back and be aware of them. I felt a little more in control (…) whereas before (…) anger just flares up (…) [but now], I have a little voice, recognizing that it's still there, but there will be a little version of myself stepping back and looking at this anger and maybe questioning it” (B1).

#### 12.2.2. Increased Awareness and Acceptance

Similarly, other participants reported that the intervention helped them by developing awareness and improving the ability to accept negative feelings and thoughts associated with depression. For example, one participant stated: “Probably it is not just about removing negative feelings or healing depression. Instead, the goal is to learn to cope with it. I would say that the condition in itself has not changed, but it feels better, because I gained another perspective” (F2).

#### 12.2.3. Increased Generosity

In addition, participants often noted that they experienced a change in their self-perception by being more generous with themselves and others. For example one participant stated: “I noticed how my pattern of thinking is actually different after the twelve weeks. So I'd say I had a different outlook or approach than usual (…) [I am] a bit more generous with people [and] myself I suppose – it's a little less dark. …I'd say [I am] a little bit more generous with myself” (B1).

#### 12.2.4. Increased Empathy and Tolerance

Participants also expressed greater empathy and tolerance. For example, one participant stated: “Perhaps it was rather new for me to change my view of other people, perhaps with more understanding, instead of rejecting them completely (…). I have a sense of life again, a certain hope (…) to get independent from others, to manage things alone, without doctor or drugs” (F3). Another participant stated the following: “I really liked the philosophy about being compassionate (…) like an expanding compassion radiating from yourself to others; and when everyone does that then all feel sort of enlightened (…) I'm literary more peaceful with myself and a little less harsh” (B4).

#### 12.2.5. Group Support

For most participants, being in a group was perceived as a positive experience. Being able to show emotions in a safe and supportive environment encouraged them to make the effort to expose themselves to the challenges of the program. One participant expressed this as follows: “It was good to hear feedback from different people about the meditation. You could kind of take that and go with it. You might hear something from someone that you did not know, and now you see a different perspective on it” (B2). Similarly, another person stated: “Everyone is sort of on the same wave length (…) if they are feeling really calm, it's like a contagious calm, and I start to feel that way [as well]” (B4).

### 12.3. Practice of Meditation

This category refers to the experience of participants with the LKM meditation exercises.

#### 12.3.1. General Evaluation

Participants typically found the intervention to be helpful: “Everything was organized dimensionally (…) there was the beloved one, the neutral person. Thinking about how this person affected you motivates you to meditate [and] gives you a reason to think about how this person affected you” (B2). However, several participants also described struggling with certain aspects of the meditation exercises: “I tend to have difficulties when it comes to sitting for a long time. I don't get restless but I feel either really sleepy or I don't feel present. Whenever it's a long meditation, it's hard for me to keep up with it” (B4).

#### 12.3.2. Focus on the “Difficult Person” 

Some participants specifically mentioned the* difficult person* metta mediation as being insightful but difficult. One person stated: “Actually, the LKM exercise* difficult person* worked after all. I focused on a person who joined the team at work, and who already had irritated me (…) but I had no difficulties to wish him good things, because I think that not everybody is always angry at me” (F4). Another person noted: “Focusing on the difficult person was a bit difficult. But I don't think it was negative; it was just going through it to look for a positive outcome” (B1).

#### 12.3.3. Experiencing Emotions

One patient stated: “Somehow, I encountered a barrier with emotions; such that I did not feel anything. I believe that I have encapsulated myself. This is something that I perceive when practicing LKM (…). Over the years, I have gotten the impression that I have lost a little bit of my inner balance. I tended to protect myself (…). I have the impression that the more distance I feel to a person, the easier it is, and the closer I am to a person, the more I question the relationship. Therefore, LKM on a neutral person or even a difficult person was even easier for me, because I thought, ‘I have everything to gain, whereas on others, in close relationships, I can only lose'” (F2).

#### 12.3.4. Meditation as Coping

One participant summarized his experience as follows: “To practice meditation is a constructive way of coping with one's world of feelings, in contrast to simply enduring [it] in a passive way” (F2). Some patients mentioned their experience with incorporating the meditation practice into their daily lives: “I am very happy that I am able to be aware (…) in another way than before, and this gives me strength and energy (…) I also discovered self-care, to give myself something good, to enjoy, and to integrate into daily life” (F3). Continuous homework practice of exercises was felt to be important but difficult to achieve.

## 13. General Discussion

The results of our study indicate that LKM results in strong decreases of depressive symptoms. Although these effects only refer to completers and are less pronounced in the sample of patients with chronic depression, the effect sizes were considerable, especially given the relatively modest effects of psychotherapy typically reported in the literature. As expected, significant increases in positive affect were observed in both samples. Although the samples differed with respect to clinical psychopathology, the results of the qualitative data analysis suggest that the intervention is positively experienced and well tolerated. Consistent with our recent model [12] and recent studies [23], our results suggest the LKM intervention leads to an increase in positive affect, an enhancement of adaptive emotional regulation strategies, an improved sense of self and others, and a greater general acceptance and emotional tolerance. However, further studies are warranted to draw more solid conclusions about the efficacy of LKM for mood disorders and the underlying mechanism of change.

This study not only points to exciting new avenues for future interventions, but also raises intriguing questions about the role of positive affect in depression and the mechanism of psychological treatment change. Positive and negative affects are not two opposite points on the same continuum. At the same time, they are not two completely unrelated constructs. Although negative affect features prominently in emotional disorders, positive affect is also an important but less investigated dimension. This suggests that some emotional disorders, including mood disorders, are associated not only with heightened negative affect, but also with lowered positive affect. The study by Carson et al. [20] reported that depressed individuals have a greater tendency to dampen positive affect and are also more apprehensive towards positive affect than nondepressed individuals. The authors noted that existing treatments are primarily focused on relieving negative affect and hypothesized that interventions that focus on positive affect might be particularly beneficial for this population. We believe that our study provides preliminary support for this notion.

Depressed mood is associated with preferential recall of negative material compared with positive material [40, 41] and rumination that leads to the dampening and reduction of positive affect [42]. Despite the evidence highlighting the importance of positive affect in depression, contemporary treatments for mood disorders (as well as most other emotional illnesses) almost exclusively focus on strategies to alleviate negative affect, whereas less attention is placed on strategies to enhance positive affect.

More recent research has shown that strategies to enhance positive affect can lead to significant improvements in overall emotional health. Consistent with this notion is the* broaden-and-build model*, which states that positive emotions loosen the influence of negative emotions on the person and at the same time broaden the behavioral repertoire by enhancing physical, social, and intellectual resources (e.g., [19]). In other words, this model assumes that positive emotions are adaptive because they provide people with an opportunity to expand on their resources and social relationships to prepare for future challenges. As a result of the frequent experience of positive affect, happy people are generally also more successful [43] and have happier and healthier lives. This is consistent with the notion that depression is associated with both heightened negative affect and a deficiency in positive affect [27]. Depending on the person's diathesis and affective style, a positive feedback loop can become established between the disorder, dysregulation, negative affect, and affective style, which leads to a chronic condition that becomes difficult to change. The model assumes that the most effective ways to treat mental disorders are by (1) targeting emotion dysregulation by promoting adaptive emotion regulation strategies; (2) downregulating negative affect and upregulating positive affect, and (3) promoting adaptive affective styles. Therefore, adding an LKM component to traditional psychotherapy (such as CBT) that primarily targets negative affect, might significantly enhance the efficacy of treating mood dysregulation, possibly by enhancing adaptive emotion regulation [23]. We also predict that such a strategy might be beneficial for treating anxiety disorders, such as PTSD, generalized anxiety disorder, and social anxiety disorder.

The limitations of this study include the small sample size, lack of an adequate control group, limitations of the assessment procedure, and high attrition rate. However, the purpose of this study was to demonstrate the feasibility of the procedure and to gather preliminary data on its efficacy. The lack of the control group is the most significant limitation. However, it is unlikely that simply the passage of time could have produced the observed effects in a group of dysthymic and chronically depressed individuals. The results of the quantitative and qualitative data analyses from two independent studies revealed highly promising findings that warrant further investigation. We recommend that future research evaluates the efficacy of CBT that incorporates the LKM component.

It should also be noted that the LKM program with its focus on mindfulness shares some similarities to traditional MBSR. Therefore, it remains uncertain whether the effects of the intervention were due to the unique LKM elements or due to the broader effects of decentering from emotions, observation of internal events without judgment, and acceptance as documented following the traditional MBSR and MBCT programs. Another avenue for future research, therefore, is to compare the processes and effects of LKM with MBSR/MBCT in controlled studies to examine the effects of loving-kindness over and above traditional mindfulness-based interventions.

## Figures and Tables

**Table 1:** Outline of loving-kindness medication (LKM) training.

Session number	Content
Session 1	(i) Introduction of instructors (ii) Logistics (meeting times, confidentiality, etc.) (iii) Psychoeducation and introduction to the principles of mindfulness and LKM meditation (iv) Defining mindfulness (nonjudgmental awareness of present moment, etc.) (v) Eating a raisin mindfully (vi) Sitting meditation with focus on breathing (vii) Homework: 5 minutes per day of sitting meditation with focus on breathing

Session 2	(i) Review of past week and homework (ii) Reviewing mindfulness concept (iii) Sitting meditation with focus on breathing (iv) Discussion of techniques, experiences, and problems (v) Closing meditation (vi) Homework: 10 minutes per day of sitting meditation with focus on breathing

Session 3	(i) Review of past week and homework (ii) Review of mindfulness concept (iii) Sitting meditation (iv) Introducing loving-kindness/metta concept: positive versus negative feelings, defining happiness as positive feelings toward others, effect of anger toward others on self, and idea of common humanity (v) LKM meditation with focus on benefactor (vi) Homework: LKM meditation while listening to scripted recorded on CD (10–15 minutes)

Session 4	(i) Review of past week and homework (ii) Review of loving-kindness/meta concept (iii) LKM meditation as before with adding focus on beloved person (iv) Homework: LKM meditation while listening to scripted recorded on CD (10–15 minutes)

Session 5	(i) Review of past week and homework (ii) Review of loving-kindness/meta concept (iii) LKM meditation as before with adding focus on self (unless it is experienced as too difficult) (iv) Homework: LKM meditation while listening to scripted recorded on CD (10–15 minutes)

Session 6	(i) Review of past week and homework (ii) Review of loving-kindness/meta concept (iii) LKM meditation as before with adding focus on neutral person (iv) Homework: LKM meditation while listening to scripted recorded on CD (10–15 minutes)

Session 7	(i) Review of past week and homework (ii) Review of loving-kindness/meta concept (iii) LKM meditation as before with adding focus on difficult person (iv) Homework: LKM meditation while listening to scripted recorded on CD (10–15 minutes)

Sessions 8–11	(i) Review of past week and homework (ii) LKM meditation as before while gradually moving to all living beings (iii) Homework: LKM meditation while listening to scripted recorded on CD (10–15 minutes)

Session 12	Sharing experiences, review of progress, discussion of problems, and planning future practices

**Table 2:** Mean summary scores and effect sizes of Study 1 (*n* = 10).

Outcome	Pretreatment		Posttreatment		*t* (9)	*p*	*d*
	M	SD	M	SD			
PANAS							
Positive	22.40	9.28	35.90	7.22	−4.53	0.001	1.63
Negative	30.90	6.38	18.10	6.52	4.51	0.001	1.98
BDI-II	21.70	6.90	3.80	3.22	9.22	<0.001	3.33
DPES							
Joy	20.50	8.58	28.90	5.86	−4.73	0.001	1.14
Contentment	14.80	6.99	23.20	6.92	−3.61	0.003	1.21
Love	21.40	3.66	26.80	3.33	−3.52	0.003	1.55
Pride	18.70	5.19	26.00	6.73	−3.63	0.003	1.22
Amusement	18.00	4.74	22.20	5.65	−2.33	0.022	0.81
Awe	23.22	8.11	32.22	6.85	−3.19	0.006	1.20
Compassion	25.00	5.64	29.40	5.52	−2.15	0.030	0.79
RRS	64.20	13.13	44.30	13.12	4.02	0.002	1.52

Note. Means (M), standard deviations (SD), *t*-test values (*t*), and Cohen's effect size *d*. The sample size is *n* = 10; PANAS = Positive and Negative Affect Schedule; BDI-II = Beck Depression Inventory-II; DPES = Differential Positive Emotions Scale; RRS = Rumination Response Scale.

**Table 3:** Mean summary scores and effect sizes of Study 2 (*n* = 8).

Outcome	Pretreatment		Posttreatment		*t* (7)	*p*	*d*
	M	SD	M	SD			
HRSD	14.50	5.88	8.75	4.23	2.26	0.029	1.12
BDI-II	32.50	7.86	19.13	6.24	8.23	<0.001	1.90
PANAS							
Positive	6.75	3.15	12.13	7.26	−2.27	0.029	0.94
Negative	15.75	8.94	9.25	5.01	2.33	0.026	0.92
RSQ	2.41	0.15	2.25	0.28	2.18	0.033	0.72
ASQ							
Concealing	3.38	0.82	3.42	0.87	0.37	0.361	0.05
Adjusting	1.75	0.53	2.10	0.68	1.73	0.064	0.58
Accepting	2.40	0.65	2.73	0.66	2.16	0.034	0.50

Note. Means (M), standard deviations (SD), *t*-test values (*t*), and Cohen's effect size *d*. The sample size is *n* = 8; HRSD = Hamilton Rating Scale for Depression; BDI-II = Beck Depression Inventory-II; PANAS = Positive and Negative Affect Schedule; RSQ Response Styles Questionnaire; ASQ = Affective Style Questionnaire.

**Table 4:** Characteristics of interviews participants.

Location/subject-ID	Age	Demographics
Boston		
B1	30	Female, white, single, graduate school, full-time employment
B2	37	Male, Latino, white, single, high school graduate, unemployed
B3	44	Male, black, single, high school graduate, part-time employed
B4	20	Female, Asian, single, partial college, student
Frankfurt		
F1	52	Female, white, Graduate school, part-time employed
F2	37	Male, white, high school graduate, full-time employment
F3	50	Female, white, Graduate school, housewife
F4	54	Male, white, high school graduate, full-time employment

Note: table shows demographic data of the eight participants who provided data for the qualitative analyses; four of them were from Study 1 (Boston, USA) and four of them from Study 2 (Frankfurt, Germany).
